# A new allele for aluminium tolerance gene in barley (*Hordeum vulgare* L.)

**DOI:** 10.1186/s12864-016-2551-3

**Published:** 2016-03-05

**Authors:** Yanling Ma, Chengdao Li, Peter R. Ryan, Sergey Shabala, Jianfeng You, Jie Liu, Chunji Liu, Meixue Zhou

**Affiliations:** Tasmanian Institute of Agriculture and School of Land and Food, University of Tasmania, P.O. Box 46, Kings Meadows, TAS 7249 Australia; Western Barley Genetics Alliance, Murdoch University, 90 South Street, Murdoch, WA 6150 Australia; CSIRO Agriculture, GPO Box 1600, Canberra, ACT 2601 Australia; CSIRO Agriculture, 306 Carmody Road, St Lucia, QLD 4067 Australia

**Keywords:** Aluminium tolerance, Barley, *HvAACT1*

## Abstract

**Background:**

Aluminium (Al) toxicity is the main factor limiting the crop production in acid soils and barley (*Hordeum vulgare* L.) is one of the most Al-sensitive of the small-grained cereals. The major gene for Al tolerance in barley is *HvAACT1* (*HvMATE*) on chromosome 4H which encodes a multidrug and toxic compound extrusion (MATE) protein. The HvAACT1 protein facilitates the Al-activated release of citrate from root apices which protects the growing cells and enables root elongation to continue. A 1 kb transposable element-like insert in the 5’ untranslated region (UTR) of *HvAACT1* is associated with increased gene expression and tolerance and a PCR-based marker is available to score for this insertion.

**Results:**

We screened a wide range of barley genotypes for Al tolerance and identified a moderately tolerant Chinese genotype named CXHKSL which did not show the typical allele in the 5’ UTR of *HvAACT1* associated with tolerance. We investigated the mechanism of Al tolerance in CXHKSL and concluded it also relies on the Al-activated release of citrate from roots. Quantitative trait loci (QTL) analysis of double haploid lines generated with CXHKSL and the Al-sensitive variety Gairdner mapped the tolerance locus to the same region as *HvAACT1* on chromosome 4H.

**Conclusions:**

Our results show that the Chinese barley genotype CXHKSL possesses a novel allele of the major Al tolerance gene *HvAACT1*.

**Electronic supplementary material:**

The online version of this article (doi:10.1186/s12864-016-2551-3) contains supplementary material, which is available to authorized users.

## Background

Aluminium (Al) is the most abundant metal in the earth crust. Al can be toxic to plants when the concentration of soluble Al increases in acidic soils (<pH 5.0) due to the formation of the phytotoxic Al^3+^ species. An early symptom of Al toxicity is the inhibition of root elongation which limits water and nutrient uptake [1–3]. The inhibition of root growth in wheat (*Triticum aestivum* L.) occurs within minutes or hours in simple hydroponic solutions [4] due to decreases in root cell division and elongation [5]. Longer treatments result in thickened roots, damaged root cap, and lesions in the epidermal and cortical tissues near the root apices [6–8]. Additional symptoms of stress in wheat and maize roots include the appearance of large swollen cortical cells near the root tip [9, 10].

An important mechanism of resistance in many species relies on the exclusion of Al from root tissues by the release of organic anions from the root apices. The organic anions such as malate and citrate chelate the harmful Al cations in the apoplast and prevent them damaging the root tissues [11–13]. Barley (*Hordeum vulgare* L.) is one of the most Al-sensitive cereal species yet it still shows genotypic variation [14]. A mechanism for Al tolerance described in barley relies on the Al-activated release of citrate from root apices. This is controlled by a single major locus called *Alp* on chromosome 4HL [15–17]. The gene underlying the *Alp* locus is *HvAACT1* which encodes a member of the multidrug and toxic compound extrusion (MATE) family [8, 14, 17, 18]. This gene has been linked with tolerance in many genotypes of barley including Murasakimochi, Dayton, Honen, WB229, Svanhals, Br2 and Brindabella [16, 19–22]. Tolerant genotypes of barley show a constitutively higher expression of *HvAACT1* in root apices than sensitive genotypes. Furthermore, constitutively over-expression of *HvAACT1* in transgenic barley and wheat plants significantly increases the Al-activated citrate efflux and their tolerance to Al in hydroponic solution and in acid soil [8].

The higher expression of *HvAACT1* in tolerant barley was recently linked to the presence of a 1023-bp transposable-element like insertion in the 5’ untranslated region (UTR) of *HvAACT1* [23]. This insertion alters the usual distribution and level of *HvAACT1* expression such that it becomes constitutively high in the root apices [11, 23]. This mutation is only found in cultivated Al-tolerant barley genotypes from East Asia where acid soils are prevalent and likely represents an important mutation that has helped the expansion of barley from the Near East where the soils are rarely acidic [23].

A PCR-based marker can be used to detect the presence or absence of the insert in the 5’ UTR of *HvAACT1* and this is a convenient method for screening barley for Al tolerance [19, 23]. The PCR product from tolerant barley genotypes is approximately 1 kb larger than the product from sensitive barley genotypes [23]. Another gene-specific marker called *HvMATE*-21 was designed to target polymorphism at the 3’ UTR of *HvAACT1* and this marker has been used successfully to score more than 50 varieties differing in Al tolerance [18]. All tolerant varieties tested possessed the 21-bp deletion compared with the sensitive varieties. Using association analysis the *HvMATE*-21 marker could explain 66.9 % of phenotypic variation for Al tolerance [18]. Meanwhile, several simple sequence repeats (SSR) markers such as Bmac310, Bmag353 and HVM03 are closely linked with tolerance and commonly used for genetic analysis [16, 20, 24].

In this study, we identified a Chinese barley variety, CXHKSL, which was moderately tolerant to acid soil (Additional file 1: Table S1) but which gave a non-standard result for the 5’ UTR *HvAACT1* marker. This indicated that Al tolerance in CXHKSL might be controlled or regulated in a different way. We investigated the Al tolerance mechanism in CXHKSL and mapped the trait using a double haploid (DH) population derived from CXHKSL and the Al-sensitive variety Gairdner.

## Methods

### Genetic materials

CXHKSL is a six-rowed Chinese variety that is tolerant to acid soils. The Al-sensitive variety, Gairdner, is an Australian malting barley. The Al tolerant variety, Dayton, was used as a control when investigating tolerance mechanisms of CXHKSL. One DH population consisting of 210 lines derived from a cross between CXHKSL and Gairdner was used for QTL mapping study.

### Al tolerance and root growth

The relative Al tolerance of the different varieties and selected double haploid lines (DHLs) were evaluated with hydroponic culture methods. Sterilized seeds were germinated in the dark for 2 days at 4 °C and then 2 days at 28 °C. Root length of the seedlings was measured and they were placed in an aerated nutrient solution containing 500 μM KNO_3_, 500 μM CaCl_2_, 500 μM NH_4_NO_3_, 150 μM MgSO_4_, 10 μM KH_2_PO4, 2 μM Fe:EDTA, 11 μM H_3_BO_3_, 2 μM MnCl_2_, 0.35 μM ZnCl_2_ and 0.2 μM CuCl_2_. For barley, Al tolerance was estimated by measuring net root length after 4 days in 0, 1, and 4 μM AlCl_3_ (pH = 4.3), respectively. Relative root length (RRL) was estimated as: (net root growth in Al treatment/net root growth in control solution) × 100 % [8]. Meanwhile, 4-day old control and 4 μM AlCl_3_ treated seedlings roots were stained with haematoxylin for 15 min and rinsed for 10 min to compare the density of Al accumulation at root apices. Haematoxylin could form a purple-red complex with Al and provides an indirect of non-complexed Al in root apices, with the intensity of staining correlated with sensitivity of Al toxicity [25].

Al tolerance was also scored using acid soil collected from the Northern Tasmania (pH = 4.3). Three seeds of each DHL and parent varieties were sown in the acid soil in each replicate. Two independent experiments including six replicates were conducted in April and June 2013, respectively. Four replications were applied in each experiment. Both root length and root morphology were used to assess Al tolerance. Root length (mm) of each seedling was measured seven days after sowing. Meanwhile, root tips were screened for the absence or presence of thickening caused by Al toxicity.

### Assaying citrate efflux and malate efflux from root apices

Seedlings were grown for 4 days in the nutrient solution described above (without added AlCl_3_). To study if the expression of *HvAACT1* need longer Al treatment duration, half of the plants of each genotype were subjected to 0.2 mM CaCl_2_ solution containing 10 μM AlCl_3_ (pH = 4.3) for overnight pre-treatment. Ten root apices (3-5 mm) with 4 replicates were excised from the same line and washed in 1 ml 0.2 mM CaCl_2_ solution (pH = 4.3) for on a platform shaker (60 rpm). After 30 min washing, 1 ml 0.2 mM CaCl_2_ solution (pH = 4.3) with 30 μM AlCl_3_ was added and shaken for 2 h at 60 rpm. The solutions were centrifuged to dryness on a rotary vacuum drier for citrate efflux detection. The enzyme assay used to determine citrate concentration is described by Wang et al. [16]. The initial citrate content in each sample was calculated from a standard curve. Malate concentration was measured with an enzyme assay as described previously [26].

### Molecular marker analysis

Three primer pairs were used to investigate allelic variation in the 5’UTR of *HvAACT1*. These were to detect the presence or absence of a ~1 kb transposon-like insertion previously described in Al-tolerant genotypes of barley. The first pair of primers was from Fujii et al. [23] with forward sequence 5’-GGTCCAACACTCTACCCTCCTT and reverse 5’-GGTGCGAG -TTGCCCCTAGCTATTA. The second pair of primers described by Bian et al. [18] was forward 5’-CTTCATTTCAACCAAGCACTCC and reverse 5’-GCTTTTGGTCGAACAAA- GTATCG. The third pair of primers was designed to amplify a slightly larger fragment that included the above two pairs of primers comprised, forward 5’-TGTCGATATGGTGCTCTT -CG and reverse 5’-AGCTCCATGACAATTCTGGG. PCR reactions were performed at 20 μl-volume including 10 μl HotstarTaq^TM^ master mix (Qiagen), 2 μl primer mix (1:1 mix of forward and reverse primers at 10 nM), 3 μl DNA template, and 5 μl H_2_O. Cycling conditions were as follows: 1 cycle of 1 min at 95 °C, 35 cycles of 1 min at 95 °C, 30 s at 60 °C, 40 s at 72 °C, and finally with an extension step of 1 min at 72 °C. All PCR reactions were run at C1000^TM^ Thermal cycler (BIO-RAD). PCR products were separated at 1 % agarose and visualized by staining with 1 % Red safe under Gel Doc^TM^ XR^+^ imagining system (BIO-RAD).

Another *HvAACT1*-specific marker, *HvMATE*-21, was used to genotype the population as well as three closely *HvAACT1*-linked SSR markers: Bmac310, Bmag353 and HVM03 [14, 16, 19]. *HvMATE*-21 was a PCR marker that detected the presence or absence of a 21-bp fragment in the 3’ UTR of *HvAACT1*. PCR reactions were carried out in a total volume of 15 μl containing 25 ~ 30 ng genomic DNA, 0.5 M of forward and reverse primers, 7.5 μl GoTaq® Hot Start Colorless Master Mix, 2X (Promega). The amplification of SSRs were performed by: 1 cycle of 3 min at 94 °C, 35 cycles of 1 min at 94 °C, 1 min at the annealing temperature 55 °C and 1 min at 72 °C, with a final extension step of 5 min at 72 °C. The PCR profiles for *HvMATE*-21 were almost the same as that for SSR markers except the annealing temperature was 60 °C. All PCR reactions were run on Mastercycler Gradient 5331 (Eppendorf AG, Germany). The PCR products were separated on 5 % denatured polyacrylamide gels and visualized by a rapid silver staining method [27].

### Isolation and sequence analysis of coding region of HvACCT1 gene in CXHKSL and Dayton

The published complete coding DNA sequence (CDS) of *HvAACT1* gene (Genebank: AB302223.1) was retrieved from the National Center for Biotechnology Information (NCBI, http://www.ncbi.nlm.nih.gov/gene) and aligned with barley reference genome data using IPK blast server (http://webblast.ipk-gatersleben.de/barley/viroblast.php). Based on the best hit sequence, a total of 4 pairs of primers (Additional file 2: Table S2) were used to amplify the whole *HvACCT1* open reading frames (ORFs). The amplified PCR products was purified and cloned with pGEM®-T Vector System (Promega). The final CDS were constructed using sequencing results from 12 independent clones (3 clones for each pair of primers). Sequence analysis was completed with software DNAMAN (version 7.0; Lynnon Biosoft, USA). The sequence data of CDS have been deposited to NCBI Genbank Database with accession number of KU725980 for variety CXHKSL and KU725981 for Dayton.

### *HvAACT1* expression

RNA was isolated from root apices (also from plants used for citrate efflux measurement) by RNeasy^TM^ plant kit (Qiagen) and purified by inclusion of RNase-free DNase (Qiagen). One microgram total RNA was used to synthesize cDNA by reverse transcriptase system (Invitrogen). 1.0 μl oligo primer was added into 11.5 μl reaction mixture including 1 μg RNA. The mixture was incubated at 70 °C for 10 min, and transferred to ice immediately. Each aliquot included 4 μl buffer, 2 μl 0.1 M DTT, 1 μl dNTP mix, and 0.5 μl superscript III Reverse Transcriptase was added into the mixture, and incubated at 42 °C for 1 h. RNA degradation step was performed by addition of 0.25 μl RNase H (Thermo Scientific™) and incubated at 37 °C f or 30 min.

Quantification real time polymerase chain reaction(RT-PCR) was run in a C1000TM Thermal cycler (BIO-RAD) with 10 μl reaction mixture containing 4.0 μl of cDNA diluted to 1:40, 5 μl of SYBR Green Jumpstart Taq Readymix (Sigma) and 1 μl primer mix (1:1 mix of forward and reverse primers at 10 nM). Three pairs of primers used to measure expression of *HvAACT1* (*HvAACT1*-forward 5’-AGCAGCCAAGACCTTGAGAA and reverse 5’-AGCAG GAATCCACAACCAAG; New-*HvAACT1*-1-forward ACGGGGCTCTACCTCTTT -GT and reverse 5’-GGCAATAGAAACACCAACAGC; New-*HvAACT1*-2-forward CTGTGTCACTC TGGCATCGT, and reverse 5’-AAGCTGCAGAACACGAGAGGT). The constitutively expressed barley glyceraldehyde-3-phosphate dehydrogenase (HvGAPDH) gene and barley homologous to eukaryotic translation elongation factor 1A (HveEF-1A) gene was used as reference genes. The sequences of primers are as follows: *HvGAPDH*-forward: 5’-GTGAGG CTGGTGCTGATTACG and reverse 5’-TGGTGCAGCTAGCATTTGAGAC, *HveEF-1A*-forward 5’-TTTCACTCTTGGTGTGAAGCAGAT and reverse 5’-GACTTCCTTCACGAT-TTCATCGTAA. Cycling conditions were 3 min at 95 °C, followed by 40 cycles at 95 °C for 10 s, 60 °C for 20 s, 68 °C for 10 s. At the end, a melting curve of the amplified fragments was produced by increasing the temperature every 0.5 °C from 60 °C to 95 °C.

### Data analysis and QTL mapping

All phenotypic data was analysed by SPSS software package (Version 20.0, IBM), including all basic statistics calculation and Chi-Square Goodness of Fit Test. For genetic linkage map analysis, the genetic distances between molecular markers were calculated using software JoinMap 4.0 [28]. The mean values of root lengths of DHLs were used to detect QTL affecting root length under Al toxicity with software MapQTL6 [29]. Interval mapping (IM) method was first used to identify the major QTL. By selecting significantly linked markers as cofactors, multiple QTL mapping (MQM) mapping method based on the multiple-QTL model was used. A set of 1000 permutations was performed to identify the LOD threshold corresponding to a genome-wide false discovery rate of 5 % (*P* < 0.05) [29].

## Results

**Fig. 1:**
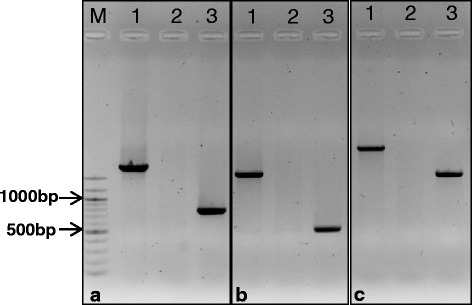
Testing presence of the 1-kb transposon-like insertion in the 5’UTR of *HvAACT1* associated with Al tolerance. Primers sets used in the PCR reactions are listed: (**a**) from Fujii et al. [23] (**b**) from Bian et al. [18] and (**c**) a new set of primers farther upstream and downstream from those used in (**a**) (see Methods). Varieties are Dayton (1), CXHKSL (2) and Gairdner (3)

A PCR-based marker is available to score for the presence or absence of the ~1 kb insert in the 5’UTR of the *HvAACT1* associated with higher expression in the root apices of Al-tolerant barley [23]. The marker generates a larger product in Al-tolerant genotypes than in sensitive genotypes. A Chinese variety named CXHKSL was identified which was tolerant to acid soil but did not show the typical marker result associated with tolerance. In fact the standard PCR reaction failed to produce any PCR band in CXHKSL while the results obtained from control varieties including the highly tolerant variety, Dayton, and sensitive variety, Gairdner, were as expected (Fig. 1a). The failure to generate a band was not related to the quality or quantity of DNA extracted from CXHKSL since other PCR reactions were successful including the *HvMATE*-21 marker which targets the 3’ UTR of *HvAACT1* (data not shown). Polymorphisms in CXHKSL may have reduced annealing temperature of one or both primers and so two additional primer pairs were designed to target this polymorphic region in the 5’ UTR of *HvAACT1* (Fig. 1b, c). One set had primers farther upstream and downstream from the first set and therefore would amplify a slightly larger product (Fig. 1c). The second set of primers (Fig. 1b) was previously described by Bian et al. [18]. Using these additional primers, PCR products of the expected size were reliably generated from Dayton and Gairdner. By contrast CXHKSL generated no products at all (Fig. 1b, c). By contrast the *HvMATE*-21 marker for CXHKSL, Dayton and Gairdner were as described by Bian et al. [18]: the tolerant genotype possessed a 21-bp deletion but sensitive genotypes did not (data not shown). The coding region of *HvAACT1* in both CXHKSL and Dayton consisted of 1668 bp which is the same as previously published CDS [15]. CXHKSL was similar to Dayton with only one nucleotide being different for *HvAACT1* coding region (Additional file 3: Figure S1) and the difference didn’t cause any changes in amino acids. Two and three SNPs were detected between the published CDS and CXHKSL and between the CDS and Dayton, respectively.

**Fig. 2:**
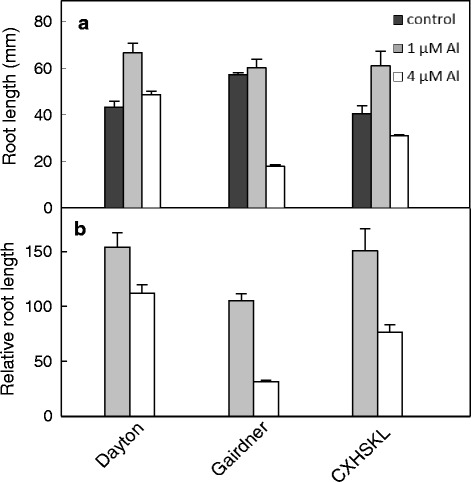
Comparison of Al tolerance in hydroponic culture. **a** Net root growth of seedlings after four days in nutrient solution containing 0, 1 or 4 μM AlCl_3_ (pH =4.3). **b** Relative root length at each Al concentration in contrast with controls. Data showed means and standard error (SE) (n = 4-7)

**Fig. 3:**
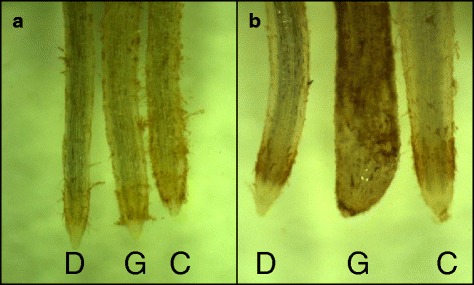
Comparison of haematoxylin staining of barley roots in hydroponic experiments following different treatments. Shown are root apices after 4 d in (**a**) control solution and (**b**) 4 μM AlCl_3_. Genotypes shown are Dayton (D), Gairdner (G) and CXHKSL (C). Darker staining indicated greater Al accumulation at the root apices

**Fig. 4:**
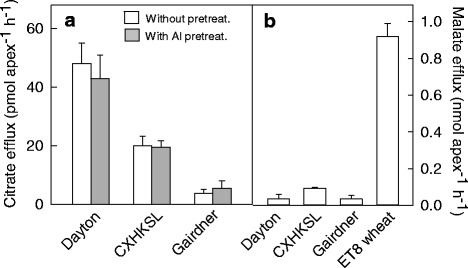
Citrate and malate efflux from the root apices of the barley varieties. **a** Citrate efflux was measured in presence of 30 μM AlCl_3_ with and without an overnight pre-treatment in 10 μM AlCl_3_. **b** Malate efflux measured in the presence of 30 μM AlCl_3_ without pre-treatment. ET8 is an Al-tolerant wheat line used as a positive control for malate efflux. Data show means and SE (n = 4)

The Al tolerance of CXHKSL was further investigated in hydroponic experiments and compared with Dayton and Gairdner. Relative root length (RRL) of CXHKSL was ~75 % after four days in 4 μM AlCl_3_ which was significantly greater than Gairdner (~30 % RRL) but less than Dayton (~110 % RRL) (Fig. 2). This ranking was similar to the Al-related tissue damage on roots in the three varieties (Fig. 3). Root apices of Gairdner became significantly thicker than the others after four days in 4 μM AlCl_3_ which is a typical symptom of Al toxicity. Haematoxylin staining was also more intense in the root apices of Gairdner than in Dayton or CXHKSL, indicating greater Al accumulation in the roots of Gairdner. The Al-dependent efflux of citrate was then measured from these barley lines. Citrate efflux from CXHKSL was less than Dayton (~40 pmol · apex^-1^ · h^-1^) but greater than Gairdner which correlated well with their relative tolerance to Al (Fig. 4a). Pre-treatment with Al prior to these measurements did not increase citrate efflux further compared with roots without pre-treatment (Fig. 4a). These results indicate that the mechanism of Al tolerance in CXHKSL is associated with Al-activated citrate efflux from roots which is consistent with previous reports for barley [14, 16]. We also investigated whether Al-activated malate release from the roots of CXHKSL was apparent but we found no indication of efflux from this in any barley lines tested (Fig. 4b). An Al-tolerant wheat line included as a positive control in this experiment showed malate efflux of 0.9 nmol apex^-1^h^-1^ which is similar to published values [26].

**Fig. 5:**
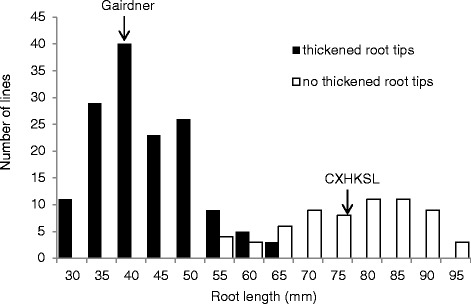
Distribution frequency of root length of 210 DHLs including parents after growth in an acid soil (pH = 4.3). Solid bars indicated genotypes with thickened root tips and white bars indicate genotypes without thickened root tips

**Fig. 6:**
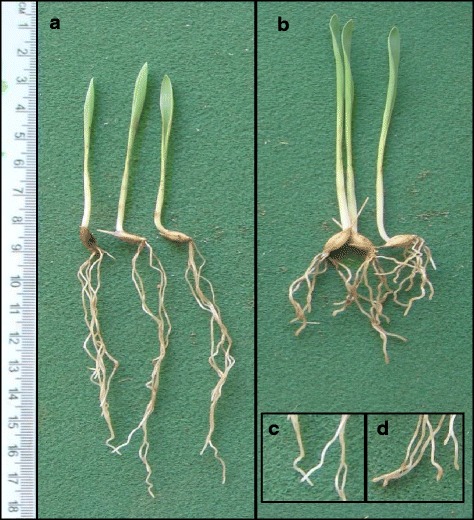
Comparison of Al tolerance of two parental varieties grown in acid soil (pH = 4.3) by root length and damages to root tips. Root length was compared between (**a**) CXHKSL and (**b**) Gairdner. Typical root apices from an Al-tolerant genotype is shown in (**c**) and typical root apices from a sensitive genotype with obvious thickening and damage is show in (**d**)

The chromosomal location of the tolerance locus in CXHKSL was investigated using a doubled haploid population generated by crossing CXHKSL and Gairdner. A total of 210 DHLs were grown in the acid soil and the roots were scored based on both root length and root tip damage (thickness). Root length of the CXHKSL parent was 75 ± 8 mm and showed no damage of root tips (Fig. 5; Fig. 6a). Root length of the Gairdner was 40 ± 5 mm and the root apices showed swelling, thickening and clear signs of damage (Fig. 5; Fig. 6b). A total of 65 DHLs showed no thickened roots or visible tissue damage (Fig. 6c) while 145 DHLs showed those strong phenotypes (Fig. 6d). When both criteria of growth and damage were used to score the population, we found that 128 DHLs had root lengths ≤ 50 mm and showed thickened root apices and 52 DHLs had root lengths ≥70 mm without visible thickening of the root apices (Fig. 5; Fig. 6)*.* The remaining 30 DHLs had root lengths from 55 to 65 mm of which 17 DHLs showed thickening of the root apices (Fig. 6d).

**Fig. 7:**
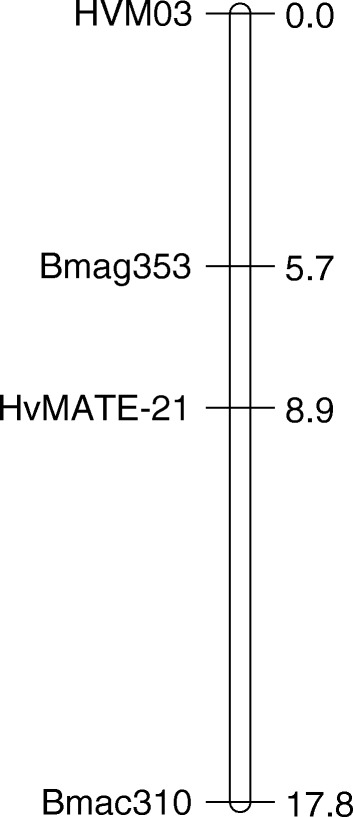
The linkage map on chromosome 4H with four molecular markers used to score 210 DHLs. Numbers on the right side represented genetic distances in unit of centiMorgan (cM)

**Fig. 8:**
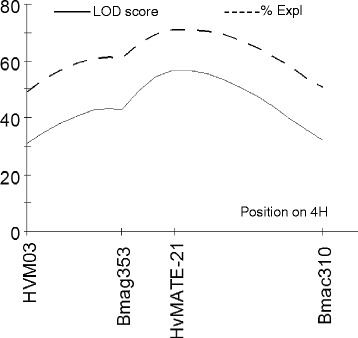
QTL detected for Al tolerance on chromosome 4H using root length variation in acid soil. The continuous line represented for the LOD score and the dashed line for phenotype variation (%) explained by each marker

Preliminary genetic analysis localised the Al tolerance phenotype in CXHKSL to a single major locus on chromosome 4H (data not shown). Therefore a more detailed genetic linkage map on chromosome 4H was generated using three SSR markers linked to Al tolerance (HVM03, Bmag353, Bmac310) as well as the *HvMATE*-21 marker which targets an 21-bp deletion in tolerant genotypes in the 3’UTR of *HvAACT1.* The map spanned a total length of 17.8 cM and the order of markers (Fig. 7) was similar to Bian et al. [18]. Analysis of root length under Al toxicity using this linkage map identified a significant QTL with a LOD score of 56.44 (Fig. 8). The closest marker, *HvMATE*-21, accounted for 71.0 % of the phenotypic variation, while Bmag353 and Bmac310 explained 61.0 % and 50.5 % of the variation respectively (Fig. 8). We conclude that Al tolerance in CXHKSL maps to the *HvAACT1* gene as reported for other tolerant barley lines. This was further tested with the *HvAACT1* 5’ UTR marker on a selection of tolerant and sensitive DHLs. All sensitive DHLs tested amplified a band similar to the Gairdner parent which is consistent with expectations. Similarly, all tolerant DHLs tested failed to produce a band which is the same as the CXHKSL parent (data not shown).

**Fig. 9:**
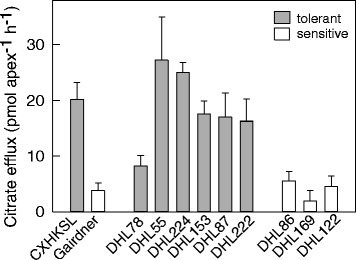
Citrate efflux from the root apices of the barley lines. Citrate efflux from CXHKSL and Gairdner as well as Al tolerant and sensitive DHLs in the presence of 30 μM AlCl_3_ without Al pre-treatment. Data showed means and SE (*n* = 4)

**Fig. 10:**
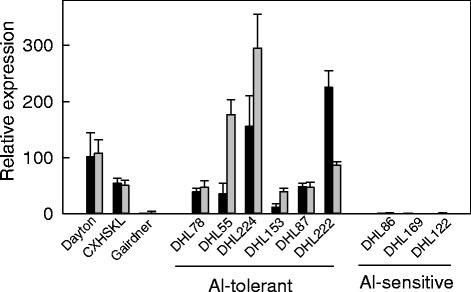
Relative expression of *HvAACT1* in Al-sensitive and Al-tolerant DH lines generated from Gairdner and CXHSKL measured with quantitative RT-PCR. The Al-tolerant variety Dayton was included. Expression of Gairdner was designated as 1.0. Data showed means of relative expression level using *HvGAPDH* (*black bars*) and *HveEF-1A* (*shaded bars*) as reference genes, and SE from three biological replicates with two technical replicates

Citrate efflux was also measured from the selection of tolerant and sensitive DHLs. Six tolerant DHLs were examined and efflux was measured from all of them. Five of these had efflux of 18 to 28 pmol · apex^-1^ · h^-1^ which was similar to the CXHKSL parent (Fig. 9). Efflux from the remaining tolerant line was lower than the other five but greater than the sensitive lines measured which were <5 pmol · apex^-1^ · h^-1^ (Fig. 9). The level of *HvAACT1* expression was also determined in selected DHLs to determine whether this was linked with the other phenotypes of tolerance and citrate efflux. These measurements used two different reference genes *HvGAPDH* and *HveEF-1A*. Expression of *HvAACT1* was detected in CXHKSL, Dayton and the tolerant DHLs tested but no expression was detected in Gairdner or the three sensitive DHLs tested (Fig. 10). These results suggest that Al-tolerance in CXHKSL is controlled by a novel allele of the *HvAACT1* gene.

## Discussion

The only mechanism of Al tolerance in barley described to date relies on the release of citrate from the root apices via the HvAACT1 transporter [14–16]. In our study, we characterised a Chinese barley variety CXHKSL which is moderately tolerant to Al stress in hydroponics and acid soil but did not generate the expected result for a standard marker that targets the 1 kb insert in the 5’UTR of *HvAACT1* correlated with Al tolerance. Dayton and Gairdner amplified fragments of expected sizes from the 5’UTR of *HvAACT1* while no PCR products were detected in CXHKSL. The determinant role of promoter variation in Al tolerance was further confirmed by the fact that only one SNP was detected for CDS of *HvAACT1* gene between CXHKSL and Dayton without causing changes in protein sequence. We showed that the Al tolerance mechanism in CXHKSL likely relies on the Al-activated efflux of citrate reported for other tolerant barley. Using a DH population generated by crossing CXHKSL and the Al-sensitive variety Gairdner and a set of markers linked with Al tolerance, we found that the tolerance locus in CXHKSL mapped to *HvAACT1*. Collectively these results suggest that CXHKSL possesses a novel allele of Al-tolerance gene *HvAACT1.*

Fuji et al. [23] reported that ~1 kb insertion in the upstream 5’UTR of *HvAACT1* alters the distribution and level of gene expression in Al-tolerant cultivars. We tried to detect the presence of this insert using the published pair of primers but no fragments were amplified in CXHKSL or the tolerant DH lines tested. However, the expression level of *HvAACT1* in CXHKSL, Dayton and Gairdner was positively correlated with the relative Al tolerance of these varieties with Dayton > CXHKSL > Gairdner. We also found that *HvAACT1* expression was higher in the tolerant DHLs than sensitive DHLs and that the marker targeting the 5’ UTR of *HvAACT1* segregated with tolerance (absence of a band in CXHKSL). This is consistent with the central role of *HvAACT1* in Al tolerance in these barley lines [23]. The absence of a PCR product with the 5’UTR *HvAACT1* marker in CXHKSL could be due to polymorphisms which prevented primer binding to the DNA. We tested this possibility by designing the additional primers from the same region but these also failed to generate a product in CXHKSL (Fig. 1c). The absence of a PCR product for this marker could also be due to a deletion in CXHKSL or the presence of a much larger insert. It is clear that polymorphisms exist in the 5’UTR of *HvAACT1* in CXHKSL compared to the published sequences for other tolerant barley [23].

The gene-specific marker *HvMATE*-21 was more efficient in predicting the phenotypic variation (71.0 % in this study, Fig. 8) under Al toxicity than other commonly-used SSR markers, Bmac310 and Bamag353 [16, 19, 20]. Meanwhile, segregation distortion occurred in the DH population which skewed the distribution of root lengths toward the sensitive parent Gairdner and away from Mendelian expectations. No significant monogenic segregation ratio 1:1(*χ*^2^ = 36.82 > *χ*^2^_0.05_ = 3.84) was observed. This distortion can occur when the generation of fertile hybrids are prevented as a result of the methods used to produce the lines [18, 30].

Al tolerance of wheat primarily relies on the Al-dependent malate efflux from root apices which is controlled by the Al-activated anion transporter encoded by the *TaALMT1* gene [31]. The closest homologue of this gene in barley is *HvALMT1* which is located on chromosome 2H and does not contribute to the Al resistance [32]. However, over-expression of *HvALMT1* gene in barley with a constitutive promoter can increase the efflux of malate and Al tolerance in barley and wheat [33]. The contribution of malate efflux to Al tolerance was investigated in CXHKSL and other tolerant and sensitive genotypes but no significant malate release was detected.

## Conclusions

In the present study, we demonstrated that CXHKSL possesses a novel allele for the major Al tolerance gene *HvAACT1* but the mechanism of tolerance is similar to other tolerant barley lines.

### Availability of supporting data

The data sets supporting the results of this article are included within the article.

## Additional files

Additional file 1: Table S1. Acid soil tolerance of barley accessions: The acid soil with pH 4.3 was collected from Northern Tasmania. Acid soil was placed in a tray (length of 2 m, width of 1 m and depth of 0.4 m) in a temperature-controlled glasshouse under a 16 h/8 h light/dark cycle (22 and 18 °C, respectively). Water level was controlled using an automatic watering system (XLSX 15 kb) Additional file 2: Table S2. List of primers used for isolations and sequencing coding regions of the *HvAACT1* gene (DOCX 14 kb) Additional file 3: Figure S1.
*HvAACT1* coding region in CXHKSL and Dayton. SNPs are shown in white background. (DOCX 149 kb)

## Abbreviations

CDS: coding DNA sequence
DH: double haploid
DHLs: double haploid lines
IM: interval mapping
INDEL: insertion-deletion
MATE: multidrug and toxic compound extrusion
MQM: multiple QTL mapping
ORF: open reading frame
QTL: quantitative trait loci
RRL: relative root length
RT-PCR: quantification real time polymerase chain reaction
SSR: simple sequence repeats
UTR: untranslated region
