# Supplementary material for: A new allele for aluminium tolerance gene in barley (Hordeum vulgare L.)
Source: BMC Genomics. 2016 Mar 5;17:186. doi: 10.1186/s12864-016-2551-3 (PMC4779196; doi:10.1186/s12864-016-2551-3)
Supplement: Additional file 2: Table S2. — List of primers used for isolations and sequencing coding regions of the HvAACT1 gene (DOCX 14 kb) [file 12864_2016_2551_MOESM2_ESM.docx]

| **Primer name** | **Primer sequences** |
| --- | --- |
| HvAACT1-g1F | 5'-GATATGTGCCACCTTCGCTG-3' |
| HvAACT1-g1R | 5'-CATCCCCAACCACTGCAAAA-3' |
| HvAACT1-g2F | 5'-TCCGGGGCTTCAAAGATACA-3' |
| HvAACT1-g2R | 5'-TGCTGCTGTGAAAGGGTCTA-3' |
| HvAACT1-g3F | 5'-GCATCAACTTCGGAGCACAA-3' |
| HvAACT1-g3R | 5'-AAGGGGACAGTGAGCTAACC-3' |
| HvAACT1-g4F | 5'-CCTCTCTCTCAGGCAAGCAT-3' |
| HvAACT1-g4R | 5'-TACCAGATGCGGGCAAATTG-3' |

**Supplementary Table S2. List of primers used for isolations and sequencing coding regions of the *HvAACT1* gene**
