# Supplementary material for: A new allele for aluminium tolerance gene in barley (Hordeum vulgare L.)
Source: BMC Genomics. 2016 Mar 5;17:186. doi: 10.1186/s12864-016-2551-3 (PMC4779196; doi:10.1186/s12864-016-2551-3)
Supplement: Additional file 3: Figure S1. — HvAACT1 coding region in CXHKSL and Dayton. SNPs are shown in white background. (DOCX 149 kb) [file 12864_2016_2551_MOESM3_ESM.docx]

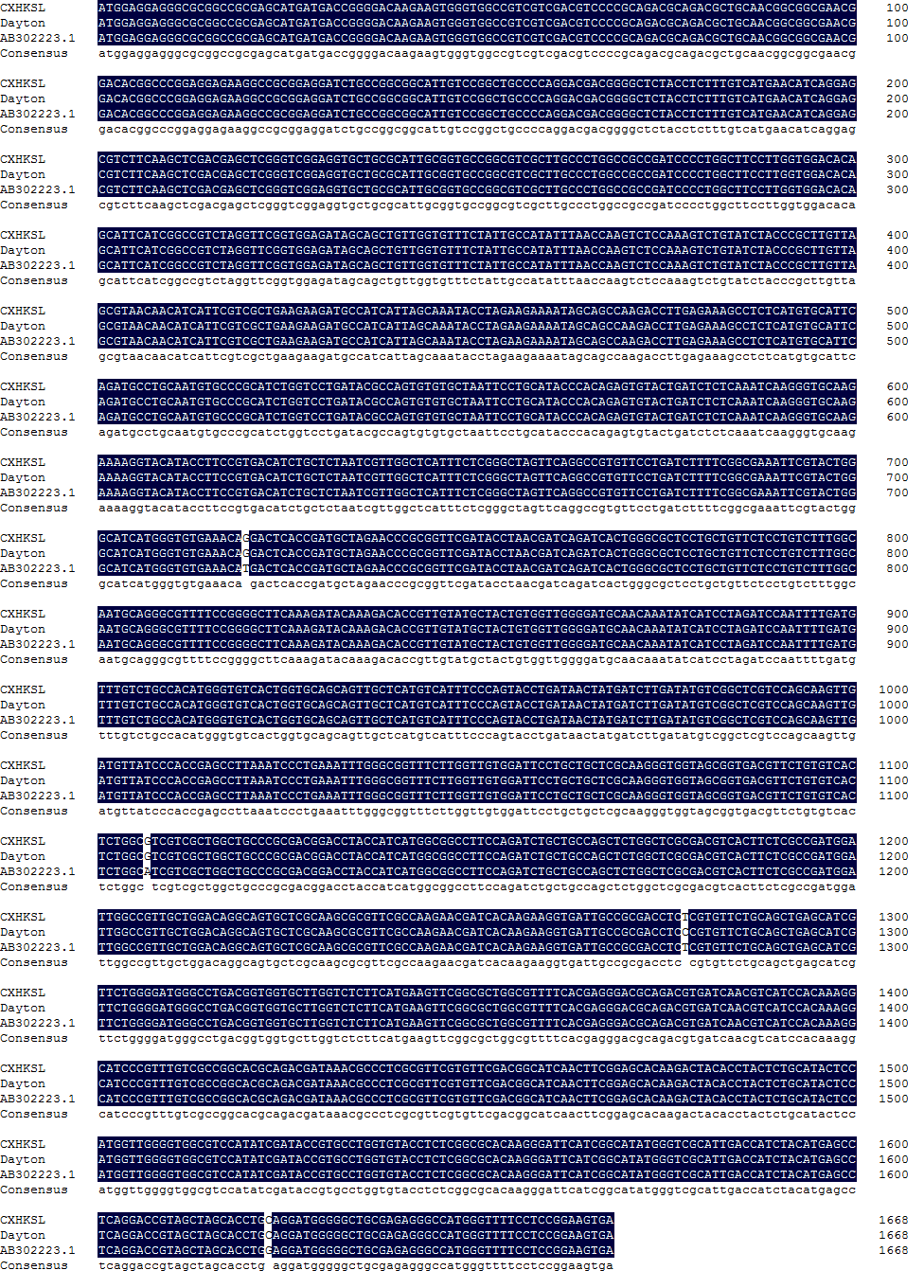


Supplementary Fig. S1 *HvAACT1* coding region in CXHKSL and Dayton. SNPs are shown in white background.
